# Influence of α-amylase, xylanase and cellulase on the rheological properties of bread dough enriched with oat bran

**DOI:** 10.1038/s41598-023-31591-y

**Published:** 2023-03-20

**Authors:** Wenjun Liu, Margaret Brennan, Dawei Tu, Charles Brennan

**Affiliations:** 1 School of Environment and Resources, Chongqing Technology and Business University, Chongqing, China; 2 Department of Wine, Food and Molecular Biosciences. Faculty of Agriculture and Life Sciences, Lincoln University, Lincoln, Christchurch, 7647 New Zealand; 3 School of Science, RMIT, Melbourne, VIC 3000 Australia

**Keywords:** Engineering, Biochemistry, Enzymes, Fluid dynamics

## Abstract

A better understanding of dough rheology during processing is crucial in the bakery industry, since quality attributes of the final product are influenced by those properties. In this study, we investigated the effects of xylanase, α-amylase and cellulase on the rheological properties of bread dough enriched in oat bran. A DoughLAB was used to measure the mixing characteristics of dough. According to the results, adding a single enzyme did not significantly affect the water absorption, development time, or stability of oat bran dough. In contrast, when blended enzymes were used at high concentrations (10, 120, and 60 ppm), the water absorption, development time, and stability of the oat bran dough were significantly reduced compared to using the single enzyme (62.1%, 7.1 and 6.6 min). It was found that combining α-amylase, xylanase and cellulase resulted in better extensibility and stickiness (16.5 mm and 60.8 g) of oat bran dough than using these enzymes individually. As a result, α-amylase, xylanase and cellulase complemented each other in determining the rheology of bread dough.

## Introduction

In the past decade, the food industry has experienced a surge of interest in functional and nutritional foods due to the exploding demand for healthier foods. During the milling of oat flour, oat bran is produced and contains a variety of dietary fibres, including water-soluble β-glucan, that can be added to food products to enhance their nutrition^1^. β-Glucan is a highly water-soluble fibre that can easily form viscous solutions, thus reducing intestinal transit time, gastric emptying time, and glucose and sterol absorption^2^. Oat β-glucan has excellent nutritional and functional properties due to its viscosity^3^. Studies have suggested that oat bran has a number of positive effects, including lowering postprandial blood glucose and decreasing insulin response and reducing serum LDL cholesterol^4–7^.

However, previous studies have illustrated that the addition of oat bran has detrimental effects on the dough rheology and final products quality. For instance, Liu et al.^8^ illustrated that the addition of 15% oat bran into Chinese steamed bread generally resulted in negative effects on rheological properties, baking performance and texture properties of final products. Additionally, the research pointed that the substitution of wheat flour with oat bran significantly decreased the bread volume accompanied by an increase in crumb firmness^9^. Xu et al.^10^ also indicated that the addition of oat bran to Chinese steamed bread led to a decrease in total moisture content and an increase in hardness and cohesiveness of bread. According to the research of Barbhai et al.^11^, the replacement of wheat flour with minor millet bran and bran rich fractions (BRF) decreased sensory acceptability of buns and muffins.

In order to improve the quality of bread incorporated with 15% oat bran, enzymes were used as individual and combination. In this study, three enzymes (α-amylase, xylanase and cellulase) were used to improve the rheological behaviour of dough during breadmaking. Fungal α-amylase is the most common enzyme used in bread making as anti-staling agents, which can randomly damage starch and reduce its water binding ability, thus increasing the gluten hydration^12,13^. Xylanase is the second most common enzyme used in food and feed, paper and pulp, textile, pharmaceuticals, which can attack the arabinoxylans (AX) backbone and break the glycosidic linkages in AX, resulting in changing the functional and physicochemical properties of AX^14^. Celluloses are widely used for extraction and clarification of fruit and vegetable juices, which can catalyze the hydrolysis of (1,4)-beta-d-glucosidic linkages in cellulose and other beta-d-glucans^15,16^. Therefore, these enzymes have potential to improve the rheological behaviour of dough incorporated with oat bran and the quality of final products^17,18^. However, there is a paucity of reports on the effects of enzymes combination, especially, the combination of cellulase, xylanase and α-amylase on the rheological properties of bread dough with 15% oat bran.

Thus, the aim of this work was to investigate the effect of α-amylase, xylanase and cellulase on the rheological properties of the bread dough with 15% content of oat bran compared to regular bread dough without oat bran.

## Materials and methods

### Materials

Wheat flour (Champion Flour Milling Ltd, Christchurch, New Zealand) and oat bran (Goodman Fielder Ltd, Auckland, New Zealand) were purchased at the local supermarket. Fungamyl 2500 SG 3.2.1.1 (2–10 ppm), Pentopan Mono BG 3.2.1.8 (20–120 ppm) and Cellulast BG 3.2.1.4 (10–60 ppm) were purchased from Novozymes Australia Pty Ltd (Novozymes, North Rocks NSW, Australia).

### Design of experiment

There were two experimental designs used to determine the effects of amylase, xylanase and cellulase individually and in combination on rheological properties of bread dough. The one-way ANOVA was performed to compare the effects of individual enzymes on the rheological properties of wheat flour dough and oat bran dough. In this study, the dosage of the xylanase, cellulase and α-amylase, recommended by Novozymes and previous research^18,19^, was added with 70 ppm, 35 ppm and 10 ppm, respectively (Supplementary Table 1).

An experimental design comprising two levels of full factorial 2^3^ was used to investigate the impact of combined enzymes on the rheological properties of oat bran dough. As reported in previous research^18,20^, each factor (α-amylase, xylanase and cellulase) at two levels (− 1, 1) provided eight different combinations of experiments, and Supplementary Table 2 shows the coded values for each factor at every level. Based on the estimated coefficients (β_i_, β_ij_ and β_ijk_), the following polynomial model was used to calculate the theoretical response function (W):

$${\text{W}}={\beta }_{0} + {\beta }_{1}{\text{A }}+ {\beta }_{2}\text{B }+ {\beta }_{3}\text{C }+ {\beta }_{12}{\text{AB} }+ {\beta }_{13}{\text{AC} }+ {\beta }_{23}{\text{BC }}+ {\beta }_{123}{\text{ABC}}$$

As a result, we obtain the total number of response variables, the global mean (β_0_), the regression coefficients for factors (β_i_), the regression coefficients for interactions (β_ij_ and β_ijk_). Three independent variables are used in this multiple linear regression model to describe the rheological property of dough that is influenced by the α-amylase, xylanase, and cellulase.

### Rheological properties of dough

The rheological characteristics of dough were measured using a DoughLAB (Perten Instruments Australia, Macquarie Park, Australia) equipped with 300 g mixing bowl following AACC 54-21.02 standard method. This instrument gives a consistent measure of the torque produced by the dough during stirring, and the principle lies being to simulate the conditions encountered during the process of baking such as mechanical shearing action. Water absorption (WA), dough development time, stability, softening, mixing tolerance index (MTI), and departure time were calculated by DoughLab software (version 1.3.0.185) on the basis of the flour weight and moisture contents. Analysis was performed in triplicate.

### Dough extension analysis

Texture analysis of dough extension was performed using a TA-XT2 Texture Analyzer (Stable Micro Systems, Surrey, UK). In order to perform extension tests, the Texture Analyser was outfitted with a Kieffer dough and gluten extensibility rig. In tension mode, resistance to extension and extensibility were determined by measuring peak force and maximum extension distances. As a regular dough, only wheat flour was used. Following previous research conducted by Liu^18,20^, wheat flour and oat bran (15 g/100 g) were combined to formulate the dough. Based on the factorial design, formulations for the optimum dough were developed. There were the following conditions: pre-test speed: 2.0 mm/s; test speed: 3.3 mm/s; post-test speed: 10.0 mm/s; distance: 75 mm; trigger force: 5 g (5 kg load cell).

### Dough stickiness

Measurement of dough stickiness was performed using a TA-XT2 Texture Analyzer (Stable Micro Systems, Surrey, UK) equipped with a Chen-Hoseney cell. The dough samples were placed into the chamber of Chen–Hoseney Dough Stickiness Cell, and then closed with a die by slowly screwing for test. Dough stickiness was determined using the Texture Expert 1.22 software under the following conditions: pre-test and test speed: 0.5 mm/s; post-test speed: 10.0 mm/s; distance: 4 mm; time: 0.1 s; trigger force: 5 g (5 kg load cell). Analysis was performed in triplicate.

### Statistical analysis

All data were treated by ANOVA and multiple regression analysis using Minitab 17 statistical software, version 17. 2. 1 (Minitab Pty Ltd, Sydney) at a significance level *p* < 0.05.

## Results and discussion

### Effect of single enzyme on the rheological properties of dough

Individual effects of enzymes on the rheological properties of wheat flour dough (regular) and dough replacing with 15% oat bran as oat bran dough are showed in Table 1. Compared to the regular dough, the oat bran dough has higher value of water absorption (67.76%), development time (9.47 min), departure time (16.13 min) and MTI (22.2 FU) accompanied by lower value of resistance to extension (21.55 g) and extensibility (11.43 mm). However, there was no significant difference in stability, softening and stickiness between regular dough and oat bran dough. According to previous research, the substitution of oat bran had a significant effect on the rheology of dough due to the disruption of starch-gluten network and high hydration properties of β-glucan^21,22^. It can be seen from Table 1, the addition of α-amylase significantly (*p* < 0.05) decreased the stability and resistance to extension of regular dough and increased the softening, MTI, extensibility and stickiness of regular dough. In terms of xylanase, the addition of xylanase increased water absorption, development time, stability, extensibility, stickiness and MTI of wheat flour dough (regular) significantly (*p* < 0.05). Additionally, the cellulase addition significantly (*p* < 0.05) increased the development time, stability, departure time, MTI, extensibility and stickiness of regular dough, and decreased both softening and resistance to extension. The effects of α-amylase, xylanase and cellulase on the rheology of regular dough have been reported and discussed in my previous study^18^.

**Table 1:** Effects of single enzyme application on the rheology of regular dough and dough incorporated with 15% oat bran.

	0		Xylanase		Amylase		Cellulase	
	Regular	Oat bran	Regular	Oat bran	Regular	Oat bran	Regular	Oat bran
WA %	63.86 ± 0.12D	67.76 ± 0.06A	65.20 ± 0.10C	67.63 ± 0.06A	63.93 ± 0.15D	66.93 ± 0.51AB	63.20 ± 0.70D	66.23 ± 0.15B
Development time (min)	2.73 ± 0.06C	9.47 ± 0.49AB	8.60 ± 0.61B	10.20 ± 0.35A	2.80 ± 0.10C	9.37 ± 0.32AB	8.76 ± 0.45B	10.20 ± 0.10A
Stability (min)	11.07 ± 0.55CDE	10.50 ± 0.36DE	18.03 ± 0.06A	11.77 ± 0.25CD	8.67 ± 0.51F	10.17 ± 0.47E	16.63 ± 0.55B	12.10 ± 0.60C
Softening (FU)	43.13 ± 1.63BC	38.63 ± 1.66CD	23.40 ± 1.75G	36.13 ± 4.44DE	60.30 ± 1.48A	48.86 ± 1.56B	31.00 ± 2.46EF	29.60 ± 0.95FG
Departure time (min)	12.67 ± 0.91E	16.13 ± 0.55CD	20.00 ± 0.01A	17.77 ± 0.15BC	10.50 ± 0.46E	14.90 ± 1.65D	18.56 ± 0.66AB	17.93 ± 0.50ABC
MTI (FU)	10.37 ± 0.92E	22.20 ± 0.92A	15.73 ± 1.16D	19.43 ± 1.13B	20.80 ± 0.20AB	21.70 ± 0.75AB	16.63 ± 1.56CD	19.33 ± 0.25BC
Resistance to extension (g)	44.59 ± 0.68A	21.55 ± 0.23F	25.73 ± 1.05E	30.55 ± 0.33D	35.37 ± 0.52C	38.23 ± 0.36B	19.96 ± 0.22G	38.44 ± 0.57B
Extensibility (mm)	31.31 ± 0.23D	11.43 ± 0.11G	39.88 ± 0.78C	13.53 ± 0.20F	45.27 ± 0.53A	15.18 ± 0.05E	43.53 ± 0.40B	15.08 ± 0.02E
Stickiness (g)	44.62 ± 0.36G	45.73 ± 0.58FG	66.72 ± 0.88A	60.25 ± 0.11B	58.43 ± 0.32C	52.38 ± 0.26E	53.86 ± 0.32D	46.42 ± 0.50F

Means ± standard deviations (n = 3). Values in the same row with different letters differ significantly (*p* < 0.05). *WA* water absorption, *MTI* mixing tolerance index, *Regular* wheat flour dough, *Bran* wheat flour dough with 15% oat bran.

In this study, we focus on how the single enzyme affects the rheological properties of dough replacing with 15% oat bran. As a result, Table 1 shows that the addition of single enzyme influenced the rheology of oat bran dough. In terms of xylanase, the addition of xylanase to oat bran dough did not significantly affect the water absorption, development time, stability, softening and departure time, whereas significantly (*p* < 0.05) decreased MTI (from 22.2 to 19.43 FU) and increased extensibility (from 11.43 to 13.53 mm) and stickiness (from 45.73 to 60.25 g). A similar result was observed by Laurikainen et al.^23^, addition of xylanase led to an increase in stickiness, but no significant effect on water absorption of wheat flour dough substituted with rye bran. Xue et al.^24^ illustrated that the addition of xylanase increased the extensibility of steamed bread dough with 15% content of wheat bran. Additionally, Flander et al.^25^ indicated that xylanase treatment increased water-extractable arabinoxylan and water-soluble polysaccharides (β-glucan), which may increase the viscosity and resistance to extension of oat dough. Therefore, this observation may be due to the change of water-soluble polysaccharides (β-glucan) content and the disruption of gluten network.

With regards to the α-amylase, the results show that there was no significant effect on water absorption, development time, stability, departure time and MTI of oat bran dough. Similar result was reported by Penella et al.^26^, single fungal α-amylase addition did not show significant effects on development and stability of dough enriched in wheat bran. Moreover, Sahnoun et al.^27^ indicated that the addition of α-amylase had minimal effect on the water absorption, development time, stability of bread dough. This observation may be attributed to the increasing content of β-glucan, which cannot be hydrolysed by α-amylase. Table 1 also shows that the addition of α-amylase significantly (*p* < 0.05) increased the softening (from 38.63 to 48.86 FU), resistance to extension (from 21.55 to 38.23 g) , extensibility (from 11.43 to 15.18 mm) and stickiness (from 45.73 to 52.38 g) of oat bran dough. Kim et al.^12^ illustrated that the addition of fungal α-amylase resulted in an increase in resistance to extension and the viscosity coefficient of dough incorporated with polished flours. According to the research of Barrera et al.^28^, the addition of α-amylase can break down the starch to dextrin and improve the fermentation behavior of bread dough. Moreover, other research has reported that the addition of α-amylase led to the dough weaker and sticky^29–32^.

In terms of cellulase, Table 1 shows that the cellulase addition decreased water absorption, softening and MTI, whereas increased stability (from 10.5 to 12.1 min), resistance to extension (from 21.55 to 38.44 g) and extensibility (from 11.43 to 15.08 mm) of oat bran dough. No significant differences in development time and stickiness was observed between oat bran dough and oat bran dough containing cellulase. Yang et al.^33^ indicated that the addition of cellulase can improve the extensibility and stability of buns dough. Additionally, Altınel and Ünal^34^ illustrated that cellulase addition presented slight and negligible changes in development time of wheat meal dough.

### Effect of enzymes combination on rheological properties of oat bran dough

**Figure 1:**
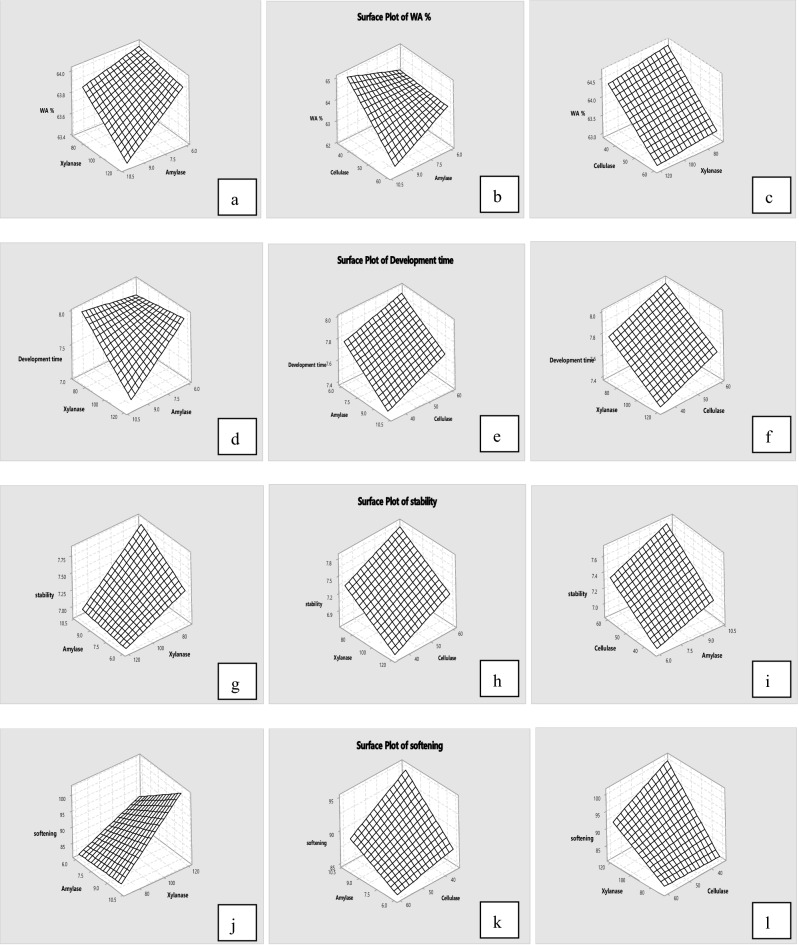

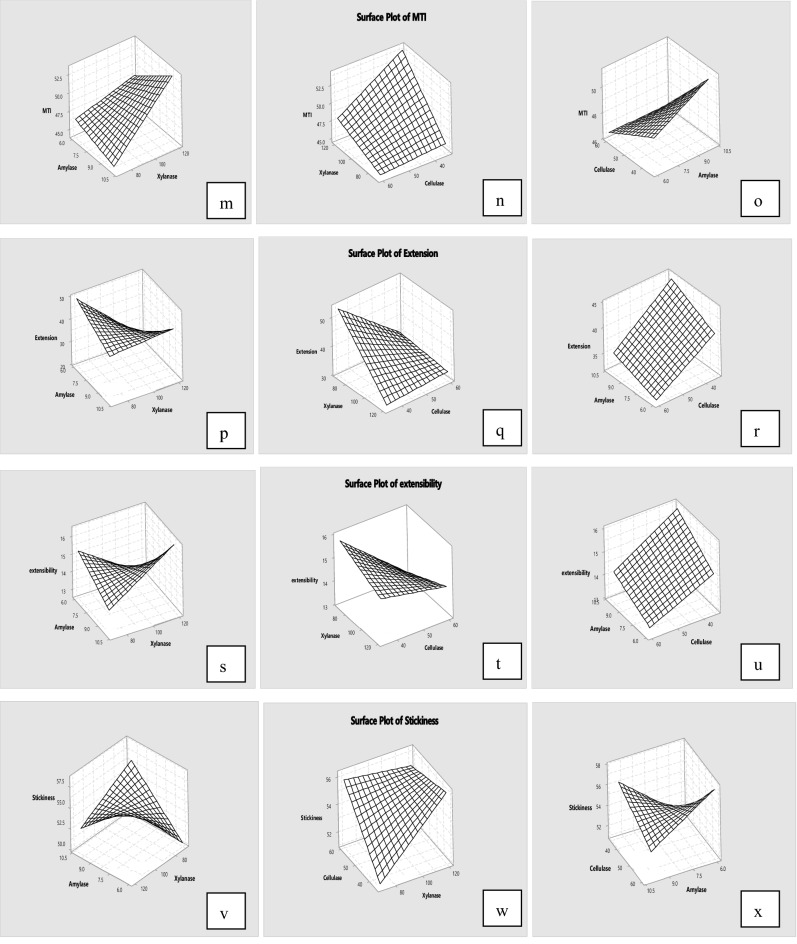
Response surface plots of oat bran dough (from **a** to **x**).

The effects of α-amylase, xylanase and cellulase combination on the rheological properties of oat bran were analysed using 2^3^ full factorial design, and analytical results are presented in Table 2. Figure 1 illustrates the interaction of α-amylase, xylanase and cellulase on parameters of oat bran dough rheology. Regression coefficients and R^2^ obtained from the full factorial design in dough rheology are presented in Table 3. Table 3 illustrates that any enzyme and interaction of the enzymes significantly (*p* < 0.05) influenced the water absorption, development time, stability, softening, departure time, MTI, resistance to extension, extensibility and stickiness of oat bran dough. Additionally, the selected coefficients represented in Table 3 were fitted to the following empirical model:

**Table 2:** Effect of enzyme combination on rheology of oat bran dough.

Blocks	A	B	C	WA %	DT (min)	Stability (min)	Softening (FU)	Departure time (min)	MTI (FU)	Extension (g)	Extensibility (mm)	Stickiness (g)
Oat bran	0	0	0	67.8	9.5	10.5	38.6	16.1	22.2	21.5	11.4	45.7
1	6	70	35	64.1	7.9	7.1	80.5	11.9	47.4	56.2	15.8	46.2
2	6	70	60	63.9	7.7	7.5	82.7	12.1	45.4	42.2	14.6	54.3
3	6	120	60	63.8	8.2	7.1	87.4	12.0	47.6	22.4	12.1	60.8
4	6	120	35	64.0	7.6	6.6	94.1	11.6	49.9	23.5	13.4	56.0
5	10	70	35	65.2	7.6	7.6	82.8	11.4	44.3	50.1	15.7	56.1
6	10	120	35	64.7	7.2	6.7	106.9	10.4	58.1	38.8	16.2	56.2
7	10	120	60	62.1	7.1	7.3	96.4	11.3	48.3	43.4	16.5	49.5
8	10	70	60	62.5	8.3	8.1	81.9	12.4	44.6	27.7	12.1	57.4

All values are means. *A (factor)* α-amylase, *B (factor)* xylanase, *C (factor)* cellulase.

**Table 3:** Estimated coefficients of the factors of the rheological properties of oat bran dough.

Coefficients	WA%	DT (min)	Stability (min)	Softening (FU)	Departure time (min)	MTI (FU)	Extension (g)	Extensibility (mm)	Stickiness (g)
Constant	63.79	7.69	7.25	89.10	11.64	48.19	38.02	14.54	54.56
A	− 0.17	− 0.16	0.16	2.90	− 0.26	NS	1.98	0.56	0.23
B	− 0.12	− 0.18	− 0.32	7.11	− 0.31	2.77	− 5.99	NS	1.06
C	− 0.71	0.11	0.25	− 1.99	0.31	− 1.72	− 4.10	− 0.73	0.92
AB	− 0.08	− 0.23	− 0.10	2.58	− 0.21	1.61	7.09	1.25	− 3.01
AC	− 0.61	NS	NS	NS	0.16	NS	NS	NS	− 2.88
BC	NS	NS	NS	− 2.30	NS	− 1.29	4.96	0.46	− 1.41
ABC	NS	− 0.18	NS	NS	NS	− 1.22	1.77	0.51	− 0.58
R^2^	97.58%	89.50%	81.11%	80.66%	95.26%	81.63%	98.92%	98.28%	99.71%

*NS* no significant effect at level (*p* < 0.05), *R*^*2*^ adjusted square coefficient (describes the percentage of variability for which the model accounts), ‘−’ negative effect; *A (factor)* α-amylase, *B (factor)* xylanase, *C (factor)* cellulase.

$${\text{W }}\,({\text{WA }} \%) =63.79 -0.17A - 0.12B - 0.71C - 0.08AB -0.61AC\, ({R}^{2}=0.97)$$

$${\text{W }} \,({\text{Development time}})=7.69-0.16A-0.18B+0.11C-0.23AB-0.18ABC \,({R}^{2}=0.89)$$

$${\text{W}}\,({\text{Stability}})=7.25+0.16A-0.32B+0.25C-0.10AB \,({R}^{2}=0.81)$$

$${\text{W}}\,({\text{Softening}})=89.10+2.90A+7.11B-1.99C+2.58AB-2.30BC \,({R}^{2}=0.81)$$

$${\text{W}}\,({\text{Departure time}})=11.64 -0.26A-0.31B+0.31C-0.21AB+0.16AC\, ({R}^{2}=0.95)$$

$${\text{W }}\,({\text{MTI}})= 48.19+2.77B -1.72C+1.61AB -1.29BC -1.22ABC\, ({R}^{2}=0.81)$$

$${\text{W}}\,({\text{Extension}})=38.02+1.98A -5.99B-4.10C+7.09AB+4.96BC+1.77ABC\, ({R}^{2}=0.98)$$

$${\text{W}}\,({\text{Extensibility}})=14.54+0.56A -0.73C+1.25AB+0.46BC+0.51ABC\, ({R}^{2}=0.98)$$

$${\text{W}}\,({\text{stickiness}})=54.56+0.23A+1.06B+0.92C-3.01AB-2.88AC-1.41BC-0.58ABC\,({R}^{2}=0.99)$$

Factors: A—α-amylase; B—xylanase; C—cellulase; AB—α-amylase*xylanase; AC—α-amylase*cellulase; BC—xylanase*cellulase; ABC—α-amylase*xylanase*cellulase.

In terms of water absorption, the enzyme combination decreased the water absorption of oat bran dough from 67.8 to 62.1% when the concentration is 10, 120 and 60 ppm. From Table 3, the α-amylase, xylanase and cellulase had a negative effect on water absorption, and the interaction of α-amylase*xylanase and α-amylase*cellulase had a negative effect as well. Compared to the single enzyme, the enzyme combinations reduced the water absorption to the minimum value when α-amylase, xylanase and cellulase were added to the highest level. Figure 1 also shows that the water absorption of oat bran dough decreased as level of enzymes increased. Similar observation was reported by Sarabhai et al.^35^, the addition of glucose oxidase, protease and xylanase combination decreased water holding capacity of millet flour dough. According to Liu et al.^18^, who suggested that the combination of α-amylase, xylanase and cellulase had a synergetic effect on the dough rheology due to the interactions among enzyme activities and their coupled reactions. Additionally, Hemalatha et al.^36^ illustrated that the mixture of α-amylase and xylanase led to a decrease in starch content and a low moisture content.

For dough development time, the addition of enzyme combination resulted in a decrease from 9.5 to 7.1 min when the enzymes added with 10, 120, 60 ppm. Both α-amylase and xylanase had a negative effect on the development time, whereas the addition of cellulase had a positive effect. The interaction of α-amylase*xylanase and α-amylase*xylanase*cellulase also showed a negative effect on development time of oat bran dough. Compared with increasing effect of single enzyme, the mixture of enzymes significantly (*p* < 0.05) decreased the development time. The similar observations were reported by Pourmohammadi and Abedi^37^ and Liu et al.^18^, who pointed out mixing time increased when the single enzyme added, whereas deceased significantly as adding enzyme combination.

With respect to stability, mixture of enzymes decreased the stability of oat bran dough from 10.5 to 6.6 min. From Table 3 and Fig. 1, both α-amylase and cellulase had a positive effect among the combination on the stability of oat bran dough, whereas xylanase showed a negative effect. Only interaction of α-amylase*xylanase had been observed a negative effect. In comparison with single enzyme, the combination enzymes decreased the stability of oat bran dough significantly. Similar observation was reported by Wang et al.^38^, the mixtures of cellulase, xylanase and lipase had a positive effect on dough gluten network. Unfortunately, there is a paucity of information regarding the effect of enzyme combination on the oat bran dough rheology. Table 3 also shows that the combination enzymes increased the softening and MTI of oat bran dough. When the concentrations of enzyme combination were added at 10, 120, 35 ppm, the value of softening and MTI were maximum, 106.9 FU and 58.1 FU respectively.

Compared with single enzyme, the blended enzymes increased extensibility and stickiness to the maximum value, 16.5 mm and 60.8 g respectively. Similar results were reported by previous study, the single enzyme or combined enzymes increased extensibility, due to the modifications in starch and arabinoxylans fractions^39,40^. Moreover, Eugenia Steffolani et al.^41^ found that dough added with enzyme mixture (α-amylase, xylanase and glucose oxidase) had the intermediate stickiness. These observations may be due to the degradation of cell wall components and higher water absorption of bran resulting in altered the water distribution among starch, protein and bran particles^42–44^. Previous research has found the dietary fibre can combine with proteins and form a matrix barrier surrounding the starch granules to reduce the enzyme activity^45,46^. However, the enzymes combination can change the fibre-protein network due to the hydrolysis mechanism of α-amylase, xylanase and cellulase.

## Conclusion

This study investigated the effects of α-amylase, xylanase and cellulase on the rheological properties of oat bran dough. As a result, the addition of single enzyme did not significantly affect the water absorption, development time and stability, whereas increased extensibility and stickiness of oat bran dough. Compared to the single enzyme, the blended enzymes can reduce the water absorption, development time and stability of oat bran dough to the minimum value when the enzyme combination were added with the high concentrations (α-amylase 10 ppm, xylanase 120 ppm and cellulase 60 ppm). In particular, the combinations of α-amylase, xylanase and cellulase can increase the extensibility and stickiness of oat bran dough to higher value than the single enzyme. Therefore, the combined enzymes were more efficient than the single enzyme in rheological properties of oat bran dough. For the baking industry, the combination enzymes can significantly shorten the formation time and stability time of dough kneading. Moreover, the combined enzymes can increase the strength and extensibility of the gluten network, thus improve the gas holding capacity of the dough. Consequently, the loaf volume, texture and flavour of bread can be improved.

## Supplementary Information

Supplementary Tables.

## Data Availability

The data used to support the findings of this study are available from the corresponding author upon request.
