# Supplementary material for: Influence of α-amylase, xylanase and cellulase on the rheological properties of bread dough enriched with oat bran
Source: Sci Rep. 2023 Mar 20;13:4534. doi: 10.1038/s41598-023-31591-y (PMC10027849; doi:10.1038/s41598-023-31591-y)
Supplement: Supplementary file 1 — Supplementary Tables. [file 41598_2023_31591_MOESM1_ESM.pdf]

Supplementary table 1 Function of enzymes (from Novozymes Biotechnology Company)

Enzymes	Component name	Activity	Fuction
Cellulast BG	endo-Glucanase	3500 EGU/g	hydrolyze (1,4)-β-D-glucosidic linkages in cellulose and other β-D-glucans
Fungamyl 2500 SG	α-Amylase	2500 FAU-F/g	hydrolyze (1,4)-α-D-glucosidic linkages in starch polysaccharides
Pentopan Mono BG	Xylanase (endo-1,4-)	2500 FXU-W/g	hydrolyze (1,4)-β-D-xylosidic linkages in xylans

EGU – Endo-Glucanase Units; FAU – Fungal Amylase Units; FXU – Fungal Xylanase Units.

Supplementary table 2 Description of experimental factors at two level

Factor	(A) α – Amylase	(B) Xylanase	(C) Cellulase
Regular	-	-	-
Control	0	0	0
F1	-1	-1	-1
F2	1	-1	-1
F3	-1	1	-1
F4	1	1	-1
F5	-1	-1	1
F6	1	-1	1
F7	-1	1	1
F8	1	1	1

Amylase (-1, 1) – (6 ppm, 10 ppm); Xylanase (-1, 1) – (70 ppm, 120 ppm); Cellulase (-1, 1) – (35 ppm, 60 ppm).
